# Pragmatic trial of an intervention to increase human papillomavirus vaccination in safety-net clinics

**DOI:** 10.1186/s12889-017-4094-1

**Published:** 2017-02-02

**Authors:** Maureen Sanderson, Juan R. Canedo, Dineo Khabele, Mary K. Fadden, Cynthia Harris, Katina Beard, Marilyn Burress, Helen Pinkerton, Cynthia Jackson, Tilicia Mayo-Gamble, Margaret K. Hargreaves, Pamela C. Hull

**Affiliations:** 1 Department of Family and Community Medicine, Meharry Medical College, 1005 Dr. D.B. Todd Jr. Blvd, Nashville, TN 37208 USA; 2 Departments of Obstetrics and Gynecology and Cancer Biology, Vanderbilt University Medical Center, Nashville, TN USA; 3 Matthew Walker Comprehensive Health Center, Nashville, TN USA; 4 Memphis Health Center, Memphis, TN USA; 5 Southside/Dodson Avenue Community Health Centers, Chattanooga, TN USA; 6 Department of Internal Medicine, Meharry Medical College, Nashville, TN USA; 7 Division of Epidemiology, Vanderbilt University Medical Center, Nashville, TN USA

**Keywords:** HPV vaccine, Hispanic, African American, Provider intervention, Safety-net clinics, Underserved

## Abstract

**Background:**

Human papillomavirus (HPV) infection has been causally linked to six cancers, and many disproportionately affect minorties. This study reports on the development and effectiveness of an intervention aimed at increasing HPV vaccine uptake among African American and Hispanic pediatric patients in safety-net clinics.

**Methods:**

Formative research, community engagement, and theory guided development of the intervention. A clustered, non-randomized controlled pragmatic trial was conducted in four clinics providing healthcare for the underserved in Tennessee, U.S., with two intervention sites and two usual care sites. Patients aged 9-18 years (*N* = 408) and their mothers (*N* = 305) enrolled, with children clustered within families. The intervention consisted of two provider/staff training sessions and provision of patient education materials, consisting of a video/flyer promoting HPV vaccine. Medical records were reviewed before/after the initial visit and after 12 months.

**Results:**

At the initial visit, provision of patient education materials and provider recommendation were higher at intervention sites versus usual care sites, and receipt of HPV vaccine was higher at intervention sites (45.4% versus 32.9%) but not significantly after adjusting for patient’s age and mother’s education. Provider recommendation, but not education materials, increased the likelihood of vaccine receipt at the initial visit, although over one-third of intervention mothers cited the flyer/video as motivating vaccination. Completion of the 3-dose series at follow-up was lower in the intervention arm.

**Conclusions:**

Future interventions should combine patient education, intensive provider/staff education, and patient reminders. Research should compare patient education focusing on HPV vaccine only versus all adolescent vaccines.

**Trial registration:**

Retrospectively registered with ClinicalTrials.gov NCT02808832, 9/12/16

**Electronic supplementary material:**

The online version of this article (doi:10.1186/s12889-017-4094-1) contains supplementary material, which is available to authorized users.

## Background

Human papillomavirus (HPV) is a known cause for cervical, vaginal, vulvar, penile, rectal, oropharyngeal and anal cancers [1–3]. Racial and ethnic disparities exist in incidence per 100,000 of HPV-associated cancers of the cervix (black 9.2, and Hispanic 9.7 versus white 7.1), vagina (black 0.6 versus white 0.4), and penis (black 0.9 versus white 0.8) [4]. Although disparities are not observed for incidence of oropharyngeal cancer, mortality for this HPV-associated cancer is higher for black men (4.8 per 100,000) compared to white men (3.7 per 100,000) [5].

Immunization guidelines for HPV vaccine indicate three doses for all 11-12 year-olds, starting as early as age 9, with catch-up vaccination for females ages 13-26 and males ages 13-21 [4, 6]. Countries with national school-based immunization programs have achieved higher uptake of HPV vaccination than other countries without school-based programs, including the U.S. [7–9]. In 2014, only 26.2% of 13-year old girls and 16.2% of 13-year old boys in the U.S. had completed the 3-dose HPV vaccine series, with the lowest female completion rates in the state of Tennessee [10]. Hispanic females in the U.S. had lower completion prior to 2013, and completion rates remain lower for African American females, compared to non-Hispanic white females [10].

Without widespread school-based provision of HPV vaccines in the U.S., healthcare provider recommendation is the strongest determinant of HPV vaccination [11, 12]. Provider-focused intervention strategies include training providers to make strong recommendations for HPV vaccination at appropriate ages [13]. Parental lack of knowledge, misinformation, and concerns about safety are common barriers to HPV vaccination [11, 12]. Patient-focused intervention strategies include education materials aimed to reduce vaccine hesitancy [13]. Very few HPV vaccine interventions have used patient education materials designed specifically for low-income African Americans and Hispanics [14, 15]. Safety-net clinics, providing primary care for underserved populations, could be leveraged to increase HPV vaccination among low-income African American and Hispanic children and reduce disparities in HPV-associated cancers.

The purpose of this study was to develop and evaluate the effectiveness of an intervention that combined provider-focused and patient-focused intervention strategies, aimed to increase HPV vaccination among African American and Hispanic pediatric patients ages 9-18 in safety-net clinics via a pragmatic trial. We hypothesized that patients at the intervention sites would be more likely to report receiving patient education materials and a provider recommendation (process measures) compared to usual care. Further, we hypothesized that patients at intervention sites would be more likely to receive the HPV vaccine at the initial visit and to complete the three-dose vaccine series by 12-months post-intervention (outcome measures) compared to usual care.

## Methods

### Setting

The study was conducted in four safety-net clinics in three cities in Tennessee, U.S. The safety-net clinics provide healthcare to low-income patients who are largely publicly insured or uninsured. Across the clinics 83-94% of patients lived in poverty, 34-61% were uninsured, and 13-50% were on Medicaid or Children’s Health Insurance Program [16]. The racial/ethnic composition of the patient populations varied, with 42-94% African American patients and 2-20% Hispanic patients.

### Formative research methods

Using a community-engaged approach and guided by the Social Ecological Model (SEM) [17, 18], the study team conducted formative research in collaboration with clinic partners and a Community Advisory Board (CAB) to develop an intervention that would be appropriate for use in safety-net clinics with racially- and ethnically-diverse, underserved patient populations. We used the SEM to examine individual, interpersonal (family), and organizational factors influencing provider behaviors and parental/patient decisions related to HPV vaccination and cervical cancer screening. In-depth interviews and focus groups were audio recorded and transcribed. ATLAS.ti software [19] was used to code emergent themes in each successive transcription using the Qualitative Description method until reaching saturation [20].

In-depth individual and group interviews (20-30 min) were collected from a purposive sample of 41 healthcare providers (physicians, physician assistants, and nurse practitioners) providing pediatric or women’s health services at the four clinic sites (additional details are in press) [21]. Focus groups were conducted with a convenience sample of African American and Hispanic female adolescents aged 16-18 years and parents, recruited from the clinic and local catchment areas. Three focus groups were conducted with mothers (*N* = 16), two groups with fathers (*N* = 14), and three groups with daughters (*N* = 15), with one each conducted in Spanish. Focus groups ranged from 3 to 9 participants. Older adolescent daughters were recruited because the study started before HPV vaccination guidelines for males were released in 2011 and was originally planned to focus on simultaneous promotion of HPV vaccination and cervical cancer screening, before guidelines were changed to begin screening at age 21. Focus group participants received $25 gift cards.

### Intervention development

We formed a CAB composed of parents and adolescents who participated in the focus groups (3 mothers, 3 fathers and 3 daughters; one Hispanic and two African American for each), 3 providers from the clinic sites and 3 community members from clinic catchment areas (one per city). The CAB met three times to assist with development of the intervention. In the first meeting, the CAB reviewed the formative research findings and gave input on the overall intervention goals, target population, key messages, intervention strategies, and format of the educational materials. Next, the study team met with the medical directors and key staff from each clinic to review the findings, share the CAB suggestions, and gather their input on the intervention plan and content.

Additional file 1: Table S1 summarizes a selected list of themes identified in analyses of the provider interview and focus group transcriptions that informed the intervention development. At the individual level, we assessed the current knowledge of mothers, fathers, and adolescent children about HPV vaccination and their preferences for receiving health education information and recommendations from their healthcare providers in the safety-net clinics about HPV vaccination. This revealed a number of barriers related to knowledge and attitudes that could be addressed in the content of the educational materials, and it pointed to several preferred options for format and mode of delivery for the CAB and CHCs to consider. At the interpersonal level, we examined how healthcare providers, mothers, fathers, and adolescent children made decisions about HPV vaccination together; overall, the mothers were the primary decision makers, with input from the adolescent increasing with age (results not shown). For this reason, we designed the educational materials with the parents as the primary audience, and the adolescents as the secondary audience. In addition, at the interpersonal level we identified the preferences of parents and teens for the interactions with their healthcare provider, which informed the plan for intervention implementation during office visits.

At the organizational level, the providers contributed valuable information about how they carry out vaccine discussions and recommendations during adolescent visits. They also identified what types of patient education materials supports would be feasible to implement in their clinic’s patient flow and that they felt would help them be able to make effective HPV vaccine recommendations to their patients. The CAB and CHC leadership considered all of these options suggested by the parents, adolescents, and providers to plan the intervention components and content. The resulting intervention included two components: 1) provider and staff training, and 2) provision of patient education materials (video and flyer).

In the second meeting, the CAB gave feedback on the text, images, and layout of the flyer; the content and text of the video script; and detailed plans for filming and editing the video. Subsequently, part of the video was filmed at one of the clinic sites, and some of the CAB members appeared in the video. In the final meeting, CAB members viewed the final educational materials and gave input on recruitment and retention strategies.

In response to guideline changes and feedback from the CAB and clinic partners, the intervention’s goal was refocused to promote HPV vaccination in both female and male adolescents (primary patient outcome), while encouraging regular cervical cancer screening for the mother accompanying them to the clinic. Modifications to the intervention based on their input included the following: included males in the study and in patient education materials; lowered the adolescent age range of eligibility from 16-18 years to 9-18 years; tailored versions of the video by ethnicity, age and readiness for HPV vaccination; and listed the six HPV-associated cancers in the education materials.

### Intervention

#### Patient education materials

The team produced an educational video, with four 3-5 min versions specific to age range (preteen 9-12 years, no mention of sexually-transmitted infection (STI); teen 13-18 years, mentioned STI) and readiness for HPV vaccination for age (ready, briefer; undecided, full version). Additional file 1: Table S2 summarizes the tailoring and topics covered in each version. The English versions targeted African Americans and the Spanish versions targeted Hispanics. The videos included images of ethnically-diverse pre-adolescents and adolescents, testimonials from an African American or Hispanic parent and physician, and images of an African American or Hispanic adolescent receiving the vaccine.

The team produced a two-sided patient information flyer in English and Spanish (Additional file 1: Exhibits S1 and S2) with a list of possible questions to ask the provider after watching the video, to help facilitate discussion. The back side listed brief highlights from the video and space for recording return appointments for the second/third vaccine doses and the Pap screening recommendation for the mother.

#### Provider and staff training

The lead investigators delivered a one-hour training session to the pediatric providers, nurses, and medical assistants at the two intervention sites. The training covered the following topics: factual information on HPV-associated cancers, HPV vaccination and cervical cancer screening guidelines; a summary of the formative research findings and input from the CAB and CHC leadership that guided tailoring of the intervention; how to follow the intervention protocol; and suggestions on what to say to patients based on the formative findings. Approximately four months after launching the intervention, the investigators delivered a refresher training. A one-hour in-service training session on childhood obesity was provided to the usual care sites.

#### Intervention protocol

After entering the exam room, the nurse/medical assistant carried out standard patient intake, then gave the flyer to the mother and asked her to answer the questions at the top (current HPV vaccination status, interest in getting the HPV vaccine, and mother’s Pap screening history). Based on the patient’s age and mother’s interest in (readiness for) the vaccine, the nurse/medical assistant played the appropriate version of the video for them to watch while waiting for the provider and instructed them to mark any questions they wanted to ask the provider. The providers were trained to make a strong recommendation for the HPV vaccine, make an appropriate recommendation for the mother regarding Pap screening, and answer any questions. If the patient received the vaccine, the nurse was to record the target return dates on the flyer and instruct the mother to make appointments at check out.

### Pragmatic trial methods

#### Trial study design

In contrast to explanatory trials that aim to assess efficacy under optimally-controlled conditions, pragmatic trials are intended to test the effectiveness of interventions in real-life conditions of routine clinical practice [22]. We conducted a clustered, non-randomized controlled pragmatic trial of an intervention in four safety-net clinic sites. The sites were matched based on racial/ethnic composition, then one site in each pair was assigned to implement the intervention, and the other two sites continued providing usual care. With more than one eligible child able to enroll per family, the pediatric patients were clustered within families. With clinic-level assignment, it was not possible to blind data collection and intervention staff. However, study participants were not told which study arm they were assigned to. The study was reviewed and approved by the Meharry Medical College and Erlanger Health System Institutional Review Boards.

#### Study sample

Inclusion criteria for adolescents were being seen as a patient at a study clinic, self-identified African American or Hispanic, male or female, aged 9-18 years, and had received no HPV vaccine or received one and was overdue for the second dose (three or more months after the first dose). Exclusion criteria were having two or more doses of HPV vaccine, mother or female guardian (referred to as “mother” henceforth) not accompanying the child, planning to move away from the city within the next 12 months, not completing the baseline assessment prior to entering the exam room, mother not providing or unable to give consent, and child not giving assent. The intervention primarily focused on initiation of the vaccine series, while previous studies have demonstrated that a major reason for non-completion is not being told that the patient needed to return for two more doses [12]. For that reason, included both non-vaccinated patients and patients who received one but were overdue for the second dose, since they may not have been previously informed of the need for more doses. Patients with two doses were excluded since they presumably would have already been aware of the need for multiple doses. Sample size calculations indicated that at least 176 participants per arm were needed to detect a 15 percentage point difference in receipt of HPV vaccine at the initial visit.

#### Recruitment

Participants were recruited into the trial between May 2013 and February 2015. Trained research assistants pre-screened all appointments for each day in the clinic systems and pre-screened walk-ins when feasible, to identify children who were potentially eligible. The research assistants attempted to approach each potentially-eligible family in the waiting room to give a study flyer, request to screen for eligibility, invite to enroll if eligible, obtain informed consent and child assent, and sign medical record release forms. In addition, to increase the volume of adolescent visits, they called existing patients and distributed flyers at health fairs to invite parents to schedule well visit appointments at the clinic, without mentioning the study.

#### Data collection

Mothers and children completed a pre-intervention questionnaire before entering the exam room as well as a post-visit questionnaire. Research assistants administered the questionnaires on tablets using a secure, computerized data management system called REDCap (Research Electronic Data Capture) [23]. Twelve months after the initial visit, research assistants contacted families to complete follow-up questionnaires via phone or REDCap online survey, which were completed by May 2016. Mothers and adolescents received $25 and $15, respectively, for completing the questionnaires each time. Medical records were abstracted, including the clinic’s records and the Tennessee Immunization Information System.

### Measures

#### HPV vaccination outcomes

Based on medical record abstractions, patients were classified on two vaccination outcomes, 1) receipt of HPV vaccine dose during the initial visit (Yes/No), and 2) HPV vaccine series completion (three or more doses) by 12 months (Yes/No).

#### Intervention process measures

Mothers reported in the post-visit questionnaire whether staff implemented the key intervention components, with comparable questions for the usual care arm. 1) Patient Education Materials Provided: In the intervention arm, mothers reported if the nurse/doctor gave them “a card/flyer with facts and questions about the HPV vaccine” and showed them “a video about the HPV vaccine.” This variable was coded as Yes if the mother reported receiving at least one, and No if neither was received. In the usual care arm, mothers were asked if the nurse/doctor gave them any printed materials about HPV vaccine (Yes/No); usual care sites did not view videos. 2) Provider Recommended Vaccine: In both arms, mothers were asked, “During your visit today, did the nurse/doctor offer the HPV vaccine for your child?” (Yes/No).

#### Intervention fidelity index

In the intervention arm, an Intervention Fidelity Index was constructed as a summed score of four items from the post-visit questionnaire where mothers reported receiving the following intervention components, with the sum ranging from 0 to 4: given patient education flyer, shown patient education video, provider answered questions about HPV vaccine, and provider offered HPV vaccine for child.

#### Reasons for vaccination and non-vaccination

In the intervention arm only, mothers whose child received the HPV vaccine during the visit were asked to indicate the reasons why they chose to get it, and mothers whose child did *not* receive the HPV vaccine during the visit were asked to indicate the reasons why they chose *not* to get it, selecting all that applied out of a list of possible reasons (see Table 4). Next, they were asked to indicate the most important reason. The reason options for each were generated from our formative research and previous studies. Two new categories were coded from the write-in responses for “other reason” for not vaccinating (“Doctor said to wait until older” and “Not in stock/Pharmacy closed”).

### Analysis

Statistical analysis was performed using SAS software, Version 9.4 [24]. Chi-square was used to compare baseline demographic characteristics between study arms. Hypotheses were tested using intention-to-treat analyses. Logistic regression analysis, accounting for clustering of adolescents within families using generalized estimating equations, was used to compare the intervention arm versus the usual care arm on the two process measures and the two vaccination outcomes.

Relative risks (RR) were reported both as crude RR and adjusted RR, to assess the impact of adjusting for relevant demographic characteristics. Next the process measures were added to the logistic regression models to estimate the effects of the process measures on vaccination outcomes. Within the intervention arm, the Intervention Fidelity Index was cross-tabulated with Receipt of HPV Vaccine Dose at Initial Visit to examine the impact of intervention fidelity on this outcome, using Chi-square. Frequencies were generated for the reasons for vaccine decisions in the intervention arm. No imputation was used for missing data. All statistical tests were two-sided using alpha < 0.05 to determine significance, while noting effects with marginal significance (alpha < 0.10) as suggestive of warranting further research.

## Results

**Fig. 1:**
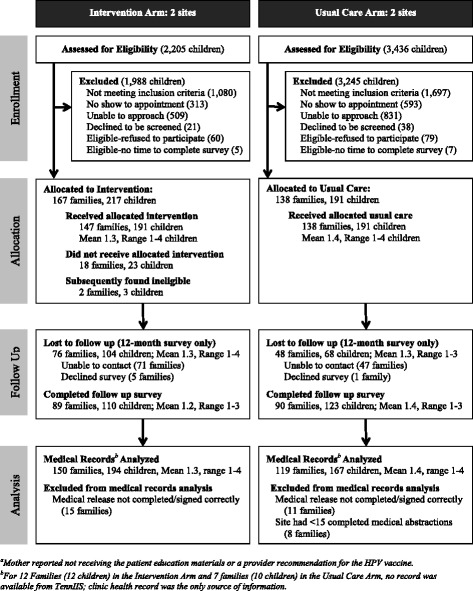
Flow diagram of study participants

Figure 1 outlines the flow of participants in the study, yielding 405 children (303 families). As shown in Fig. 1 the refusal rate, based on children who either declined to be screened or refused to participate, differed significantly between the intervention arm (81/298 = 27.2%) and the usual care arm (117/308 = 38.0%) (*p* = .005). Medical records were successfully abstracted for 361 children (89.1%). Since one clinic site in the usual care arm only successfully enrolled 17 children, resulting in fewer than 15 medical abstractions, this site was excluded from the present analysis, which used medical abstraction data. Including these cases does not change the results reported below. Thus, the analytic sample included 194 children (150 families) in the intervention arm and 167 children (119 families) in the usual care arm. Table 1 presents the baseline characteristics of the participants by study arm. Mothers’ education (*p* = .03) and child’s age (*p* < .01) significantly differed by study arm, so these variables were included in adjusted analyses.

**Table 1:** Demographic characteristics of participants with medical record data by study arm

	Intervention	Usual care	
Variable	*N* (%)	*N* (%)	*P*-value
Mothers	150 Families	119 Families	
*Number of Enrolled Children*			0.77
One	112 (74.7)	83 (69.8)	
Two	30 (20.0)	27 (22.7)	
Three	6 (4.0)	6 (5.0)	
Four	2 (1.3)	3 (2.5)	
*Age*			0.14
25-29	11 (7.9)	12 (10.8)	
30-39	65 (46.8)	64 (57.7)	
40-49	46 (33.1)	23 (20.7)	
50 and older	17 (12.2)	12 (10.8)	
Missing	11	8	
*Race/Ethnicity*			0.85
African American	131 (87.3)	103 (86.6)	
Hispanic	19 (12.7)	16 (13.4)	
*Family Monthly Income*			0.11
Less than $1,000	41 (27.7)	31 (26.7)	
$1,000-$1,999	68 (46.0)	39 (33.6)	
$2,000 and over	27 (18.2)	32 (27.6)	
Don’t Know	12 (8.1)	14 (12.1)	
Missing	2	3	
*Educational Level*			0.03
Less than high school	21 (14.3)	20 (17.1)	
Completed high school	65 (44.2)	32 (27.4)	
Some college or technical school	42 (28.6)	39 (32.3)	
Graduated from college or more	19 (12.9)	26 (22.2)	
Missing	3	2	
Children	194 Children	167 Children	
*Gender*			0.32
Boy	92 (47.4)	88 (52.7)	
Girl	102 (52.6)	79 (47.3)	
*Age*			<0.01
9-12	100 (51.6)	118 (70.6)	
13-15	47 (24.2)	35 (21.0)	
16-18	47 (24.2)	14 (8.4)	
*Race/Ethnicity*			0.82
African American	174 (89.7)	151 (90.4)	
Hispanic	20 (10.3)	16 (9.6)	
*Insurance Status*			0.08
None	36 (18.9)	17 (10.6)	
Public	136 (71.6)	123 (76.9)	
Private/Other	18 (9.5)	20 (12.5)	
Don’t know/Missing	4	7	

Table 2 presents the process measures and patient outcomes by study arm. Approximately 90% of patients in the intervention arm received patient education materials about HPV vaccine and a provider recommendation for HPV vaccine during the initial visit, compared to 33.8% (adjusted RR = 2.88, CI 2.06-4.01) and 50.3% (adjusted RR = 1.70, CI 1.38-2.10), respectively, in the usual care arm. In the intervention arm, 45.4% of patients received the HPV vaccine during the initial visit, compared to 32.9% in the usual care arm, which was not significant after adjusting for mother’s education and child’s age (adjusted RR = 1.18, CI 0.87-1.60). Completion of the three-dose vaccine series by 12-month follow-up was significantly lower in the intervention arm versus usual care arm (12.4% versus 18.0%; adjusted RR = 0.50, CI = 0.29-0.88).

**Table 2:** Process measures and HPV vaccination outcomes by study arm

	Process measure: patient education materials provided^a^		Process measure: provider recommended HPV vaccine		Patient outcome: receipt of HPV vaccine dose at initial visit		Patient outcome: completion of 3 doses at 12-month follow up	
	No	Yes	No	Yes	No	Yes	No	Yes
Study Arm	N^b^ (%)	*N* (%)	*N* (%)	*N* (%)	*N* (%)	*N* (%)	*N* (%)	*N* (%)
Usual Care (UC) Arm	92 (66.2)	47 (33.8)	82 (49.7)	83 (50.3)	112 (67.1)	55 (32.9)	137 (82.0)	30 (18.0)
Intervention Arm	15 (7.9)	174 (92.1)	20 (10.8)	166 (89.2)	106 (54.6)	88 (45.4)	170 (87.6)	24 (12.4)
Crude RR^c^ (95% CI)^d^	2.72 (1.97-3.76)		1.77 (1.44-2.19)		1.38 (1.03-1.85)		0.69 (0.40-1.17)	
Adjusted RR^e^ (95% CI)^d^	2.88 (2.06-4.01)		1.70 (1.38-2.10)		1.18 (0.87-1.60)		0.50 (0.29-0.88)	

^a^Patient Education Materials included the intervention video and/or card in the intervention arm, or any printed materials about HPV vaccine in the usual care arm

^b^ *N* Number of children

^c^ *RR* Relative risk

^d^ *CI* Confidence interval

^e^ *RR* Relative risk adjusted for mother’s educational level and child’s age

Table 3 reports multivariate models to assess the effect of the process measures on vaccination outcomes. Provision of patient education materials did not increase the likelihood of vaccination at the initial visit or completion by follow-up. Patients who received a provider recommendation during the initial visit were four times more likely to be vaccinated (adjusted RR = 4.08, CI 2.17-7.66), but were not more likely to complete by follow-up. The adjusted intervention effects in the multivariate models mirrored those in Table 2. Within the intervention arm, the Intervention Fidelity Index suggested a positive trend toward greater likelihood of vaccination at the initial visit, but it was only marginally significant (*p* = 0.07) (See Additional file 1: Table S3).

**Table 3:** Multivariate models of effects of intervention and process measures on HPV vaccination outcomes

	Receipt of HPV vaccine dose at initial visit		Completion of 3 doses at 12-month follow up	
	Adjusted RR^b^	(95% CI)^c^	Adjusted RR^b^	(95% CI)^c^
Intervention (usual care-reference)	0.86	(0.61-1.21)	0.48	(0.25-0.92)
Patient education materials provided^a^	1.13	(0.76-1.68)	0.93	(0.49-1.78)
Provider recommended vaccine	4.08	(2.17-7.66)	1.44	(0.68-3.05)
Mother’s educational level	0.92	(0.78-1.08)	0.82	(0.61-1.09)
Child’s age	1.07	(1.02-1.12)	1.19	(1.09-1.30)

^a^Patient Education Materials included the intervention video and/or card in the intervention arm, or any printed materials about HPV vaccine in the usual care arm

^b^ *RR* relative risk adjusted for other variables in the model

^c^ *CI* confidence interval

The most common mother-reported reasons for accepting HPV vaccination at the initial clinic visit were protecting child from cancer, preventing serious diseases, video/flyer gave information needed, and protecting child from HPV via future sexual partners (Table 4). Three of these were also cited as the most important reasons. Provider recommendation and provider answering questions were the next most common reasons.

**Table 4:** Mother-reported reasons for receiving or not receiving the HPV vaccination at initial clinic visit, intervention arm only

	All reasons selected^a^ %	Most important reason %
Reasons for Receiving HPV Vaccine^b^	*n* = 76	*n* = 71
Protect child from cancer	57.9	39.4
Vaccines important to prevent serious diseases	44.7	11.3
Video and flyer gave information needed	38.2	5.6
Protect child from HPV via future sexual partners	35.5	18.3
Doctor/nurse recommended	31.6	0.0
Doctor/nurse answered questions	30.3	1.4
Better to be safe than sorry	27.6	5.6
Help my child live long and happy life	25.0	5.6
Believe vaccine is safe	23.7	2.8
Don't want to regret it later if child gets cancer	18.4	2.8
Cancer runs in my family	7.9	1.4
Vaccine has had no serious side effects	7.9	0.0
Other reason/Don't know	2.6	5.6
Reasons for Not Receiving HPV Vaccine^c^	*n* = 71	*n* = 59
Want more time to think about it	25.4	5.1
Want more information about vaccine	18.3	6.8
Doctor said contraindicated due to fever/illness	16.9	15.3
Child is too young for this vaccine	15.5	8.5
Worry about safety and side effects	15.5	15.3
Child doesn't need it because not sexually active	15.5	10.2
Didn't have time today	9.9	10.2
Vaccine is too new	8.5	1.7
Doctor/nurse did not offer it today	7.0	5.1
May encourage child to become sexually active	4.2	3.4
Doctor said to wait until older	4.2	5.1
Not in stock/Pharmacy closed	4.2	1.7
Want to discuss it with family members first	2.8	1.7
Child doesn't need it because risk of cancer is low	2.8	0.0
Never heard of vaccine and don't know what it is	1.4	0.0
Don't trust drug companies	1.4	1.7
Child afraid of shots	0.0	0.0
Child doesn't need it because already sexually active	0.0	0.0
Don’t trust my doctor/nurse	0.0	0.0
Costs too much	0.0	0.0
Other reason/Don’t know	11.3	8.5

*Note*: Wording from original questionnaire items has been abbreviated for this table

^a^ Mothers could select more than one response

^b^Among mothers who completed the post-visit survey and whose child received HPV vaccine at the initial visit

^c^Among mothers who completed the post-visit survey and whose child did *not* receive HPV vaccine at the initial visit

The most common reasons for *not* receiving the vaccine included wanting more time to think about it, wanting more information, contraindication, child being too young, concern about safety/side effects, and feeling the child did not need it due to not being sexually active. When asked to select the most important reason, the most common were contraindication and concern about safety/side effects.

## Discussion

Based on the SEM and formative research with providers, parents, and adolescents, we used a community-engaged approach to develop an intervention aimed to increase HPV vaccination and evaluated its implementation in a pragmatic trial in safety-net clinics. Only two previous studies have tested provider- or patient-focused interventions designed for African American, Hispanic, or low-income adolescent patients [14, 15].

Receipt of an HPV vaccine dose during the initial visit was 12.5 percentage points higher in the intervention versus usual care arm, but the difference was not significant after adjusting for patient’s age and mother’s education. In other words, part of the difference across arms was due to a difference in patient characteristics. Adolescent age has been positively associated with HPV vaccine uptake in numerous studies [11].

The intervention successfully improved the targeted changes for providers and staff, with more frequent provision of patient education materials and provider recommendation versus usual care. In turn, provider recommendation increased the likelihood of vaccine receipt at the initial visit, although provision of the patient education materials did not. However, intervention arm mothers reported the video/flyer as the third most common reason for obtaining the vaccine (38%), suggesting they were influential for some parents in making a decision to vaccinate.

Several interventions including provider training on guidelines and strategies for increasing HPV vaccination have had an effect in increasing uptake [25–27]. However, only one study has compared the impact of different message framing approaches on HPV vaccination outcomes [28]. Subsequent to designing the intervention materials for this study, our team conducted another study using a social marketing approach and found that parents who viewed the HPV vaccine as unique from other vaccines were more hesitant to get it for their children [29]. Our findings suggested that parents may be more willing to accept HPV vaccine when it is presented as part of the overall adolescent vaccine platform that is recommended for 11-12 year-olds (HPV, Tetanus-diphtheria-pertussis, meningococcal vaccines), rather than singling out HPV vaccine for focused education, particularly for younger adolescents. Thus, our patient education materials for the present study that focused solely on the HPV vaccine could have had an unintended effect of increasing hesitancy for some parents. More research is needed to compare these different approaches and whether they should be tailored for different age ranges. Even while bundling recommended adolescent vaccines together, it is important for providers and educational materials to explain that HPV vaccination does not eliminate the need for cervical cancer screening for females starting at age 21.

The intervention did not improve completion of the three-dose series at 12-month follow up. The intervention training encouraged providers to order follow-up appointments and for staff to write the dates on the patient flyer as a reminder. However, a patient reminder system was not employed to systematically send reminders to parents about scheduled appointments. Several studies have shown patient reminders to be effective in increasing series completion [30–32]. Future clinic-based interventions should consider including automated reminder systems to enhance series completion.

### Limitations and challenges

The main study limitations were the small number of clinics and patients and the non-random assignment of clinics to study arms. The wide CIs argue for cautious interpretation, and we did not have sufficient power to examine interaction effects by gender or ethnicity. Due to the nature of pragmatic trials being conducted in real-life settings, randomization is not always feasible, and the setting is not highly controlled [22]. However, pragmatic trials such as these contribute valuable information about the effectiveness of interventions in contexts of routine clinical practice. This study highlighted mothers’ education and child’s age as important patient-population characteristics that should be addressed in future studies, either by tailoring interventions based on these characteristics, by matching similar clinics prior to randomization, or by controlling for these factors in analyses. Although we attempted to match similar clinics prior to randomization by including safety-net clinics only, the influence of mother’s education appears to have persisted. The study experienced several challenges in collaborating with safety-net clinics as study sites with on-site research staff, including staff turnover, frequent no-shows or late arrivals to appointments, and immunizations being out of stock or unavailable due to temporarily suspension of Vaccines for Children eligibility. Finally, while we gathered the formative data and community-engaged input from African American and Hispanic patients of safety-net clinics and tailored the intervention materials to match language and ethnic background, we did not seek to identify or incorporate differences in deeper cultural aspects such as beliefs, values, and norms into the intervention. Future research may consider the utility of a more extensive culturally-targeted approach.

### Conclusion

Safety-net clinics serve diverse, low-income patient populatoins. This study demonstrated the feasibility of training safety-net clinic staff and providers to utilize tailored patient education materials developed through a community-engaged process and offer the HPV vaccine to patients. However, implementation of these changes did not lead to improved vaccine uptake or completion compared to usual care after adjusting for age and education. Future reseach should examine more intensive provider/staff education, include patient reminders, and compare the effectiveness of patient education materials that focus solely on HPV vaccine versus all adolescent vaccines.

## Conclusions

This study demonstrated the feasibility of training safety-net clinic staff and providers to utilize tailored patient education materials and recommend the HPV vaccine to patients. Future reseach should examine more intensive provider/staff education, include patient reminders, and compare patient education materials that focus on HPV vaccine versus all adolescent vaccines.

## Additional file

Additional file 1: Supplementary Material. (DOC 1220 kb)

## Abbreviations

CAB: Community Advisory Board
HPV: Human papillomavirus
RR: Relative risk
SEM: Social ecological model
STI: Sexually transmitted infection
