# Supplementary material for: Pragmatic trial of an intervention to increase human papillomavirus vaccination in safety-net clinics
Source: BMC Public Health. 2017 Feb 2;17:158. doi: 10.1186/s12889-017-4094-1 (PMC5290601; doi:10.1186/s12889-017-4094-1)
Supplement: Supplementary file 1 — Supplementary Material. (DOC 1220 kb) [file 12889_2017_4094_MOESM1_ESM.doc]

**Additional file**

**Table S1. Formative Research: Key Themes that Informed Intervention Development**

| ***Providers:* Current HPV vaccine recommendation practices** |
| --- |
| Most offered the HPV vaccine to their patients at the recommended ages |
| Most checked for needed immunizations at all visits, not only annual well visits |
| ***Providers:* Perceived barriers to HPV vaccine initiation (first dose)** |
| Lack of knowledge and misinformation |
| Concern about possible side effects |
| Concern about newness of vaccine |
| Link with sexually-transmitted infections (STIs) |
| Fear of promoting sexual activity |
| Perceived costs (i.e., not knowing it is available free through age 18) |
| Believing can wait because child was not sexually active |
| ***Providers:* Perceived barriers to HPV vaccine completion (three doses)** |
| Provider not communicating the need for three doses clearly |
| Not complying with or understanding instructions |
| Lack of time, inconvenience |
| Forgetting |
| ***Parents and Adolescents:* Perceived barriers to HPV vaccine initiation (first dose)** |
| Provider not recommending HPV vaccine |
| Lack of knowledge |
| ***Parents and Adolescents:* Perceived barriers to HPV vaccine completion (three doses)** |
| Provider/staff not communicating the need for three doses clearly |
| Forgetting, lack of reminders |
| ***Parents and Adolescents:* Knowledge** |
| Low - Causes of cervical cancer |
| Low - Link between HPV infection and cervical cancer |
| Low - How the HPV vaccine provides protection |
| Moderate - HPV and cervical cancer generally related to unprotected sex or multiple partners |
| **Recommendations for development of educational materials** |
| ***Providers, Parents, and Adolescents:*** |
| Interactive materials using plain language and visual aids |
| Intentional communication about need to return for second and third doses (e.g., appointments, reminders) |
| ***Providers:*** |
| Video to watch while waiting for provider in exam room |
| Colorful printed materials at a low reading level |
| Include images of diverse people who look like the patients |
| Materials in both English and Spanish |
| Tailor information to the age of the patient |
| Downplay sexual transmission of HPV infection |
| Start as early as possible |
| ***Parents and Adolescents:*** |
| Not brochure alone (will throw it away not read) |
| Printed material together with provider explaining verbally and answering questions |
| ***Parents:*** |
| Want providers to offer vaccine proactively, not wait for the parents to ask for it |
| Clear explanation of link between HPV infection and cancer |
| Clear explanation of role of HPV vaccine for cancer prevention |
| ***Adolescents:*** |
| Digital media tools such as smartphone applications and games with knowledge quizzes |

Table S2. Tailoring of Video Content by Ethnicity, Age, and Readiness for HPV Vaccination

| **Topics Covered** | **Pre-Teen Ready** | **Pre-Teen Undecided** | **Teen Ready** | **Teen Undecided** |
| --- | --- | --- | --- | --- |
| What is HPV? (6 HPV-associated cancers) | X | X | X | X |
| How common is HPV infection? |  | X | X^a^ | X |
| How is HPV spread? |  |  | X | X |
| How is HPV infection linked to cancer? |  | X |  | X |
| How can we prevent HPV infection? | X | X |  | X |
| Who should get vaccinated? | X | X | X | X |
| Why get the vaccine now? |  | X^b^ |  | X |
| Is the HPV vaccine safe? |  | X |  | X |
| Preventing cervical cancer  (Pap screening for women ages 21+) | X | X | X | X |
| **Testimonials** |  |  |  |  |
| Parent who vaccinated own child  English: African American parent Spanish: Hispanic parent | X | X | X | X |
| Physician recommending HPV vaccine English: African American physician  Spanish: Hispanic physician | X | X | X | X |

^a^Briefer language

^b^Modified language

Note: The Pre-Teen Undecided versions of the video can be viewed at: <https://www.youtube.com/watch?v=DSgvwx5B0_c> (English) and <https://www.youtube.com/watch?v=epGQbwENi30> (Spanish)

**Table S3. Association of Intervention Fidelity Index on HPV Receipt, Intervention Arm Only**

|  | **Receipt of HPV Vaccine  Dose at Initial Visit** | |
| --- | --- | --- |
|  | **No** | **Yes** |
|  | **N (%)** | **N (%)** |
| **Intervention Fidelity Index**^a^ |  |  |
| **0** | 2 (1.9) | 1 (1.1) |
| **1** | 3 (2.9) | 0 (0.0) |
| **2** | 16 (15.2) | 7 (8.1) |
| **3** | 20 (19.0) | 10 (11.5) |
| **4** | 61 (61.0) | 69 (79.3) |
| **Missing** | 1 | 1 |
|  |  | |
| **Chi-Square P-value** | 0.07 | |

^a^Fidelity Index is a summed score of whether participant reported receiving each of the following intervention components, with the sum ranging from 0 to 4: 1) Given flyer, 2) Shown video, 3) Provider answered questions, 4) Provider recommended vaccine.

**Exhibit S1. Patient Education Flyer in English (front and back)**

**
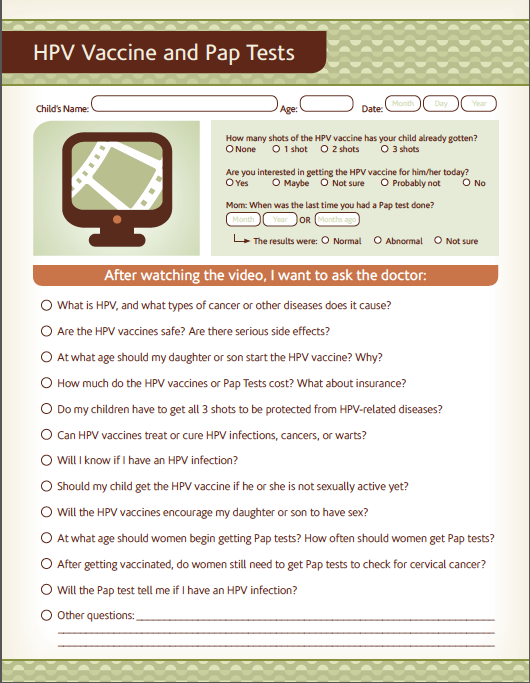
**

**
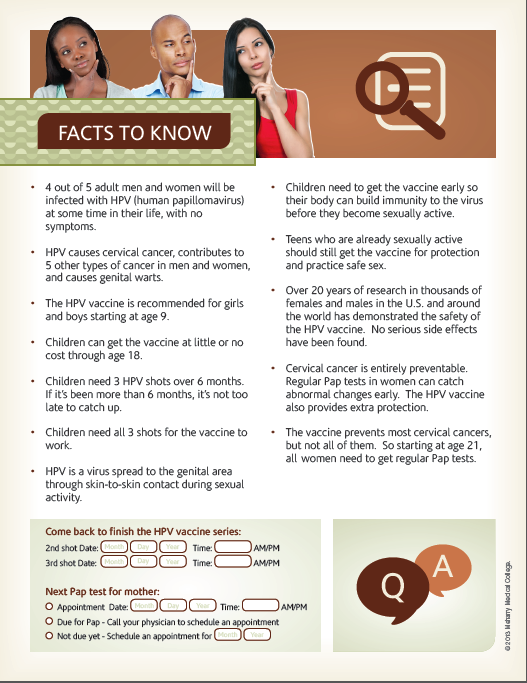
**

**Exhibit S2. Patient Education Flyer in Spanish (front and back)**

**
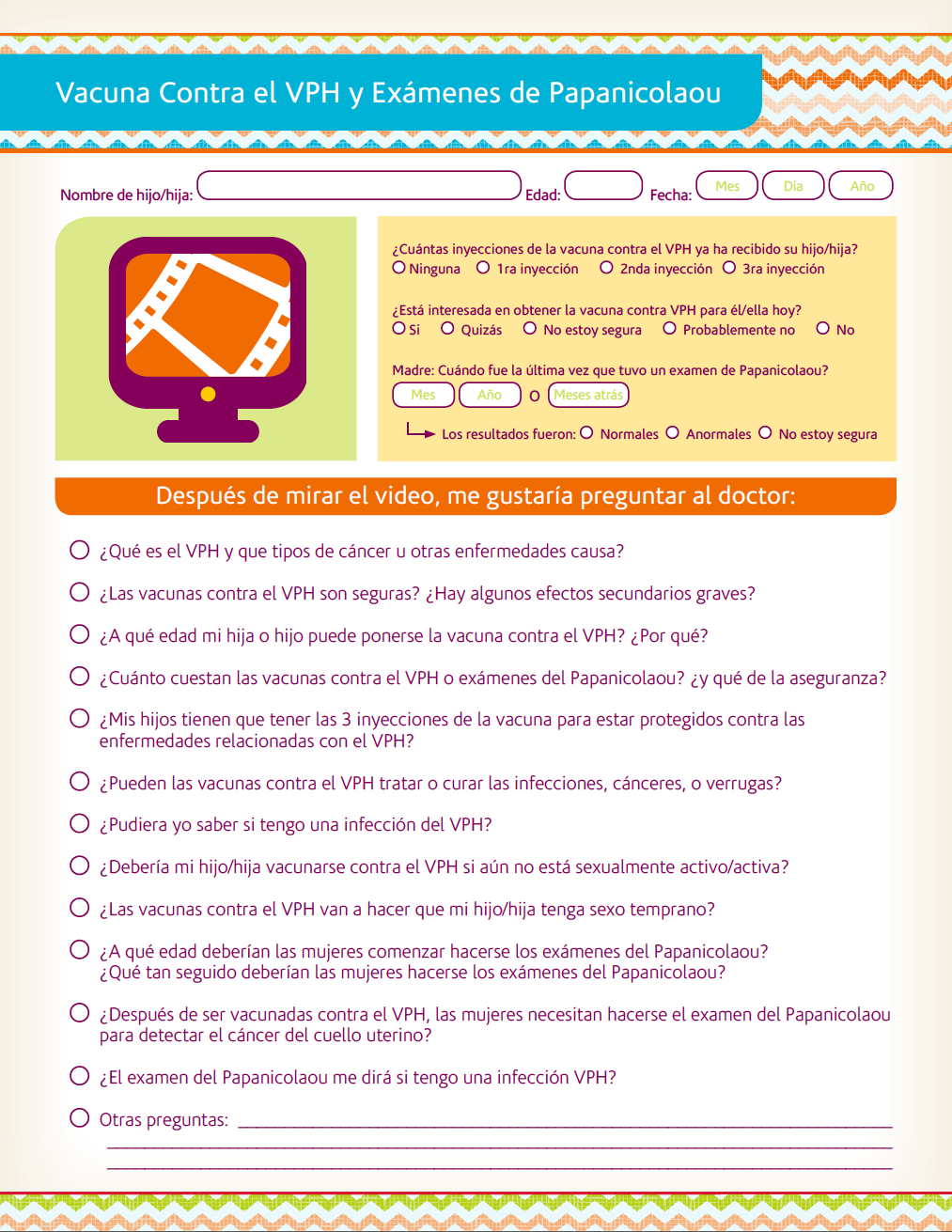
**

**
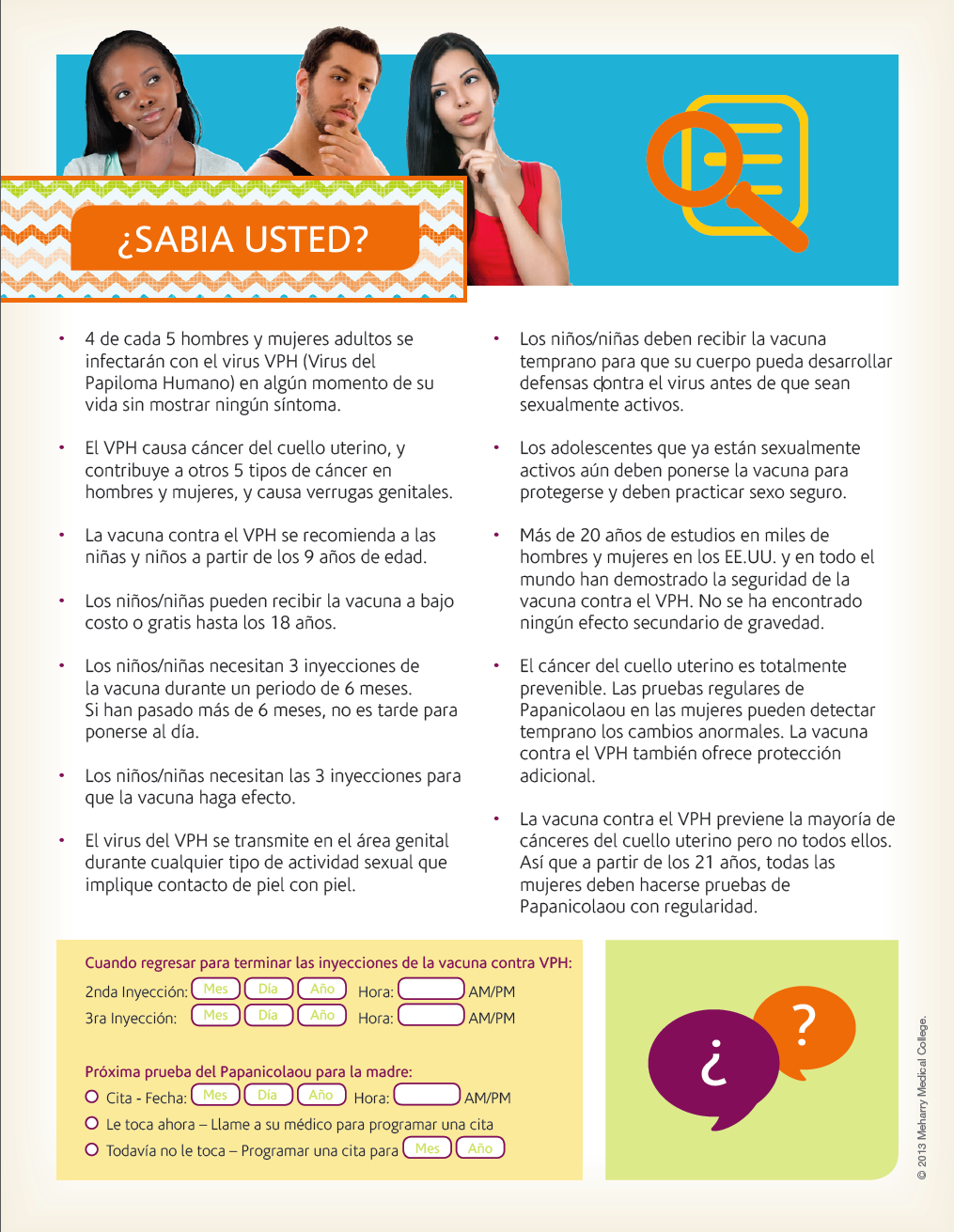
**
